# Efficacy of internal and external teat sealants on cure and new infection risk in dry-off protocols for Holstein cows

**DOI:** 10.3168/jdsc.2024-0574

**Published:** 2024-05-09

**Authors:** J.A.A. McArt, M. Wieland

**Affiliations:** Department of Population Medicine and Diagnostic Sciences, College of Veterinary Medicine, Cornell University, Ithaca, NY 14853

## Abstract

Internal teat sealant products have been used alone or in combination with antibiotic dry cow treatment to prevent new IMI over the dry period in dairy cows. Conversely, knowledge about the efficacy of external teat sealants in the prevention of IMI is scarce. The objective of our study was to investigate the effect of 2 different teat sealants, one internal teat sealant and one external teat sealant, on the (1) risk of new IMI during the dry period, (2) risk of IMI cure, (3) first test day linear SCS, (4) first test day milk yield, (5) incidence of farm-diagnosed clinical mastitis ≤30 DIM, and (6) incidence of culling ≤30 DIM. In a randomized clinical trial, Holstein cows (n = 1,378) from one commercial dairy were assigned to treatment and control groups. At dry-off, cows in the treatment groups received an antibiotic dry cow treatment in combination with either an internal teat sealant (INT) or a single application of an external teat sealant (EXT). Control (CON) cows received the antibiotic dry cow treatment alone. Data on linear SCS from the last DHI test day before dry-off and the first test after calving, first test day milk yield, and the occurrence of farm-diagnosed clinical mastitis and culling ≤30 DIM were obtained from the farm management program. New IMI and cure of IMI during the dry period were calculated. Linear SCS (mean ± SD) at first test day after calving differed among groups and was 3.2 ± 2.2 in CON, 2.8 ± 2.0 in INT, and 3.0 ± 2.1 in EXT groups. The risk of new IMI differed among groups and was 30.2% for CON cows, 18.2% for INT cows, and 22.6% for EXT cows. A Poisson regression analysis revealed that, compared with CON cows, the risks of new IMI were 40% lower for INT cows (risk ratio [RR] = 0.60, 95% CI = 0.41 to 0.88) and 25% lower for EXT cows (RR = 0.75, 95% CI = 0.52 to 1.07). By contrast, no meaningful differences were documented for cure risk, clinical mastitis risk during the first 30 DIM, or culling risk within the first 30 DIM. In summary, cows dried off with an INT in combination with antibiotic dry cow treatment had a lower linear SCS at first test day after calving and reduced risk of new IMI than cows dried off with an EXT in addition to antibiotic dry cow treatment or cows dried off using antibiotic dry cow treatment alone. Further, we found supporting evidence that cows dried off with an EXT in addition to antibiotic dry cow treatment might have an advantage in reduced new IMI over cows dried off with an antibiotic dry cow treatment alone.

## Highlights

• Teat sealants can provide an artificial barrier and mitigate pathogen invasion.
• Cows treated with INT+AB have a reduced risk of new IMI compared with AB alone.
• Cows treated with EXT+AB might have a lower risk of new IMI compared with AB alone.

## Graphical Abstract

**Figure:**
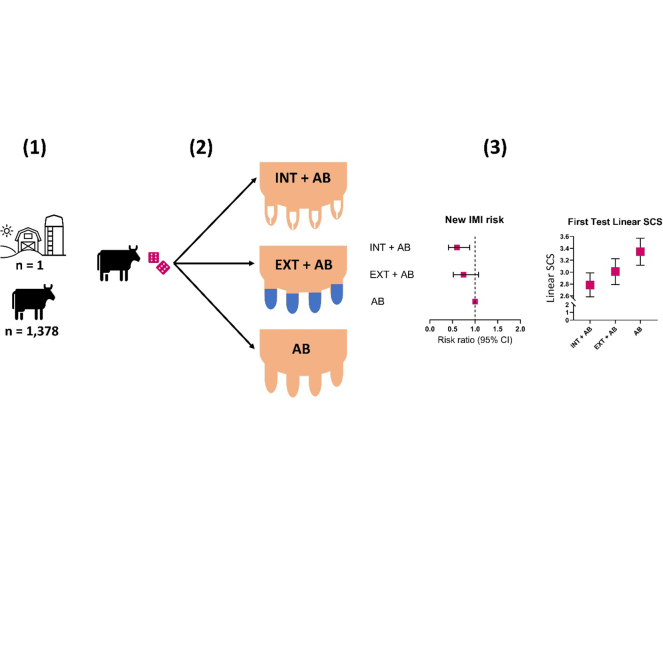
**Summary:** Our objective was to describe the efficacy of an internal and external teat sealant administered at dry-off in improving early-lactation udder health. Cows received an antibiotic dry cow treatment (AB) alone or in combination with either an internal teat sealant (INT+AB) or a single application of an external teat sealant (EXT+AB). Cows dried off with an internal teat sealant in combination with antibiotic dry cow treatment had a reduced risk of new intramammary infections (IMI) and lower linear somatic cell score (SCS) at the first test after calving than those dried off with an external teat sealant in addition to antibiotic dry cow treatment or cows dried off using antibiotic dry cow treatment alone. Further, we found evidence that cows dried off with an external teat sealant in addition to antibiotic dry cow treatment might have reduced new IMI compared with cows dried off with an antibiotic dry cow treatment alone.

Mastitis is one of the most important diseases in dairy cows with negative impacts on animal welfare and profitability of the dairy industry (Ruegg, 2017). Most mastitis cases are caused by IMI with pathogens entering the mammary gland through the teat canal (O'Shea, 1987). The dry period has long been recognized as an important factor in the dynamics of IMI with higher IMI risk shortly after dry-off and before calving (Bradley and Green, 2004). One part of the natural defense mechanisms of the nonlactating quarter against the invasion of mastitis causing pathogens is the formation of a keratin plug within the teat canal. However, the formation of this protective barrier is thought to vary among cows and takes several weeks to form, leaving the mammary gland susceptible to IMI (Williamson et al., 1995; Dingwell et al., 2004). The industry has therefore sought methods to provide an artificial barrier to seal the teat canal and mitigate the risk of IMI during the dry period (Meaney, 1977; Williamson et al., 1995; Woolford et al., 1998). The most commonly used teat sealant contains a bismuth subnitrate base and is injected into the teat canal and teat cistern at the time of dry-off. This internal teat sealant (**INT**) was developed in 1977 (Meaney, 1977) and has been repeatedly shown to reduce the risk of IMI during the dry period when used without or in combination with antibiotic dry cow treatment (Rabiee and Lean, 2013). Another form of artificial barriers is external teat sealants (**EXT**). After application, EXT generate a polymer-based film over the teat that prevents the entrance of pathogens into the teat canal (Godden et al., 2003). However, in contrast to INT, the efficacy of EXT in the prevention of IMI during the dry period is less well known.

The objective of our study was to describe the effect of 2 different teat sealants, one INT and one EXT, on IMI during the dry period. We hypothesized that cows treated with INT at dry-off, in combination with antibiotic dry cow treatment, would have lower risks of new IMI during the dry period compared with cows treated with EXT in addition to antibiotic dry cow treatment or with antibiotic dry cow treatment alone. We further hypothesized that cows treated with either INT at dry-off, in combination with antibiotic dry cow treatment, would have a higher risk of IMI cure, lower first DHI test linear SCS, higher first DHI test day milk yield, lower risk of clinical mastitis ≤30 DIM after calving, and lower culling risk ≤30 DIM as compared with those treated with EXT in combination with antibiotic dry cow treatment or antibiotic dry cow treatment alone.

We enrolled cows into a randomized clinical trial from one herd in Cayuga County, New York, between November 2017 and May 2018. The farm, herd veterinarian (J. McArt, Cornell Ambulatory and Production Medicine Clinic, Ithaca, NY), and milk quality veterinarian (M. Wieland, Quality Milk Production Services, Ithaca, NY) developed this study in response to a lack of commercially available INT. The study was reviewed by the Cornell University Institutional Animal Care and Use Committee and determined that it outlined procedures performed on live vertebrate animals that were clinically required for the animals and their condition, and that these procedures did not meet the regulatory description for use of animals for research, teaching, or testing; thus, the study was exempted from Institutional Animal Care and Use review. The study was approved by the Cornell University Veterinary Clinical Studies Committee following an ethical and scientific review (protocol no. 21319–02). We prepared our manuscript following the REFLECT reporting guidelines for randomized controlled trials for livestock and food safety (O'Connor et al., 2010). An a priori sample size was not calculated for the study. Before study initiation, the farm and consulting veterinarians decided to enroll 1,500 cows.

During the study period, the farm milked approximately 3,900 cows thrice daily in a 100-stall rotary parlor (DeLaval, Tumba, Sweden) and averaged 39.5 kg milk per cow per d. The 12-mo average values of udder health indices (calculated as the mean values from the last 12 test days before the start of the study) were mean test day SCC, 230,000 cells/mL; IMI at dry-off (defined as a linear somatic cell score [**LS**] at the DHI test before dry-off [**LSDRY**] ≥4), 27%; and dry cow new IMI (defined as a LSDRY <4 and a LS at the first DHI test LS after calving [**LS1**] ≥4), 15%. All lactating cows were eligible for enrollment at dry-off. Cows were randomized into 1 of 3 treatment groups assigned by the farm's herd management software program (DairyComp 305, Valley Agricultural Software, Tulare, CA), where all functional quarters within a cow were treated in the same manner with (1) 1 tube of intramammary dry-cow antibiotic (cloxacillin benzathine, Dry-Clox; Boehringer Ingelheim Animal Health) administered into each teat (**CON** group), (2) 1 tube of intramammary dry-cow antibiotic followed by 1 tube of INT (bismuth subnitrate, Orbeseal, Zoetis) administered into each teat (INT group), or (3) 1 tube of intramammary dry-cow antibiotic followed by a single dipping of each teat into an EXT (EXT group; 0.1% triclosan, T-HEXX, Huvepharma). Before the start of the study, the farm's dry-off protocol was identical to the INT group.

Dry-off occurred once per week on Tuesday at 0800 h. Primiparous cows were dried off between 209 and 219 d of gestation and multiparous cows between 225 and 235 d of gestation; a subset of additional cows were selected to be dried off before 209 d of gestation by the farm management team due to low milk production. Cows were loaded onto the rotary parlor until all cows were on the platform and parlor rotation stopped. Seven trained farm employees dried off cows with each person drying off one cow before continuing around the platform to the next available cow; farm employees were not blinded to group assignment. Cow identification was recorded via ear radiofrequency ID tags read by the farm's electronic parlor system (DeLaval) and confirmed by visual ear tag matching; the employee that dried off each cow was recorded. Before dry-off, red bands were placed on each hindlimb just above the dewclaws, and teat ends were scrubbed with 70% isopropyl alcohol-soaked gauze. Intramammary antibiotic and INT tubes were administered following label instructions. For cows receiving EXT, each teat was dipped once, slowly, using a 90-mL paper cup (Dixie Cup Corporation, Wilson, PA).

It took approximately 60 min to dry off all cows on a given week. Following the dry-off procedure of the last cow, rotation of the parlor platform was resumed, and cows were walked to a dry pen approximately 0.25 km away from the parlor. Time from dry-off of the first and last cow until entrance to the far-off dry pen was estimated at 80 and 20 min, respectively. Cows remained in the far-off dry pen until 3 wk before expected calving when they were moved to 1 of 2 close-up pens. After calving, cows were milked once in a 4-stall herringbone parlor in the maternity barn within 8 h of calving and then moved to an early-lactation pen. Cows stayed in the early-lactation pen for 14 to 21 d at which time they were moved to 1 of 4 high production pens. All pens but 1 were equipped with deep-bedded stalls; 1 close-up pen had mattresses. All cows were bedded daily with recycled manure solids with 6% quick lime (calcium hydroxide).

In accordance with previous studies, we used test day milk SCC as a proxy for IMI (Schukken et al., 2003; Lipkens et al., 2019). Outcomes of interest included the (1) risk of new IMI, defined as LSDRY <4.0 and a LS1 ≥4; (2) risk of IMI cure, defined as LSDRY ≥4.0 and a LS1 <4.0; (3) LS1; (4) first DHI test day milk yield; (5) the incidence of farm-diagnosed clinical mastitis ≤30 DIM; and (6) the incidence of culling (died or sold) ≤30 DIM. Data were collected from the farm's computer records at the conclusion of the study. All data were used directly from farm records except parity at calving, which was grouped into parity = 2, parity = 3, and parity ≥ 4, and milk yield at first DHI test, which was converted from pounds into kilograms per day. Data associated with first DHI test were included only for cows sampled between 5 and 35 DIM on the day of testing. Somatic cell count data were based on monthly DHI testing, which occurred on the first Wednesday of each month and was performed by Dairy One (Ithaca, NY). Clinical mastitis was diagnosed by farm employees based on visual detection of a red, swollen quarter(s) or presence of milk garget during the stripping step of the udder preparation routine.

Statistical analyses were performed in SAS version 9.4 (SAS Institute Inc.) with graph creation using GraphPad Prism (version 10.2.1, GraphPad Software). Descriptive statistics were completed using PROC FREQ, PROC MEANS, and PROC GLM. We chose to conduct univariable analyses over multivariable analyses as treatment groups were tested as farm-level dry-off management strategies, and the analyses of the study population revealed no meaningful differences among the possible confounders parity, dry period length, LSDRY, DIM at first test after calving, and presence of nonlactating quarters. We used Fisher's exact tests to evaluate differences in risk of new IMI, risk of IMI cure, and incidences of clinical mastitis and culling among groups via PROC FREQ. For outcomes with a difference among treatment groups, PROC GENMOD was used to compare the risks via Poisson regression using the Tukey-Kramer method to adjust for multiple comparisons; cows were assumed to have equivalent periods at risk. Differences in LS1 and first DHI test day milk among groups were analyzed via one-way ANOVA using PROC GLM and adjusting for multiple comparisons using the Tukey-Kramer method.

A total of 1,378 cows were enrolled in our study, which was less than the a priori selected sample size of 1,500. However, due to the renewed commercial availability of internal teat sealant, the farm chose to end enrollment. One cow was randomized inappropriately, leaving n = 1,377 for analysis. Final treatment group enrollment included 454 CON cows, 462 INT cows, and 461 EXT cows. Twenty-five cows were missing LSDRY data, 249 cows were missing LS1 data, and 178 cows were sampled for their first DHI test between 0 and 4 DIM. These cows remained in the study but were excluded for analyses in which these data were required.

Days dry ranged from 5 to 170 and did not differ among groups (*P* = 0.65), with groups having a mean (± SD) of 61 ± 22 d (CON), 60 ± 20 d (INT), and 60 ± 21 d (EXT). There was also no meaningful difference in parity group at calving among treatments (*P* = 0.65) with a median parity (range) of 3 (2 to 7) for the CON group, 3 (2 to 8) for the INT group, and 3 (2 to 9) for the EXT group. Similarly, mean DIM at first DHI test also did not differ among groups (*P* = 0.39) with a range of 5 to 35 d and a mean (± SD) of 17 ± 8 DIM for each group with n = 375, n = 390, and n = 378 for CON, INT, and EXT groups, respectively. Frequency distributions of nonlactating quarters were not different among groups (*P* = 0.11) and were CON, 50/454 (11.0%); INT, 42/462 (9.1%); and EXT, 62/461 (13.5%). Linear score at dry-off for the remaining 1,352 cows ranged from 0.1 to 9.6 and did not differ among groups (*P* = 0.72), with groups having a mean (± SD) LSDRY of 3.3 ± 1.6 (CON; n = 447), 3.3 ± 1.6 (INT; n = 451), and 3.4 ± 1.6 (EXT; n = 454). A total of 454/1,352 (33.6%) had a LSDRY ≥4 (CON, 160/447 [35.8%]; INT, 150/451 [33.3%]; EXT, 144/454 [31.7%]; *P* = 0.43).

There were 758 cows dried off with LSDRY <4.0 that had LS1 data. Of these cows, 178 (23.5%) had LS1 ≥4.0 and were classified as a new IMI. The risk of new IMI differed among treatment groups (*P* = 0.006; Figure 1A) and was 30.2% for CON cows (73/242), 18.2% for INT cows (47/259), and 22.6% for EXT cows (58/257). Compared with cows in the CON group, the RR (95% CI) of a new IMI were 0.60 (0.41 to 0.88; *P* = 0.006) for cows in group INT and 0.75 (0.52 to 1.07; *P* = 0.14) for cows in group EXT. When comparing cows that received INT to EXT, the RR was 0.80 (95% CI = 0.54 to 1.20; *P* = 0.41). There were 356 cows dried off with LSDRY ≥4.0 that had LS1 data. Of these cows, 227 (63.8%) had LS1 <4.0 and were classified as a cure of IMI. The risk of IMI cure did not differ among treatment groups (*P* = 0.29) and was 58.2% for CON cows (71/122), 67.2% for INT cows (82/122), and 66.1% for EXT cows (74/112).

**Figure 1:**
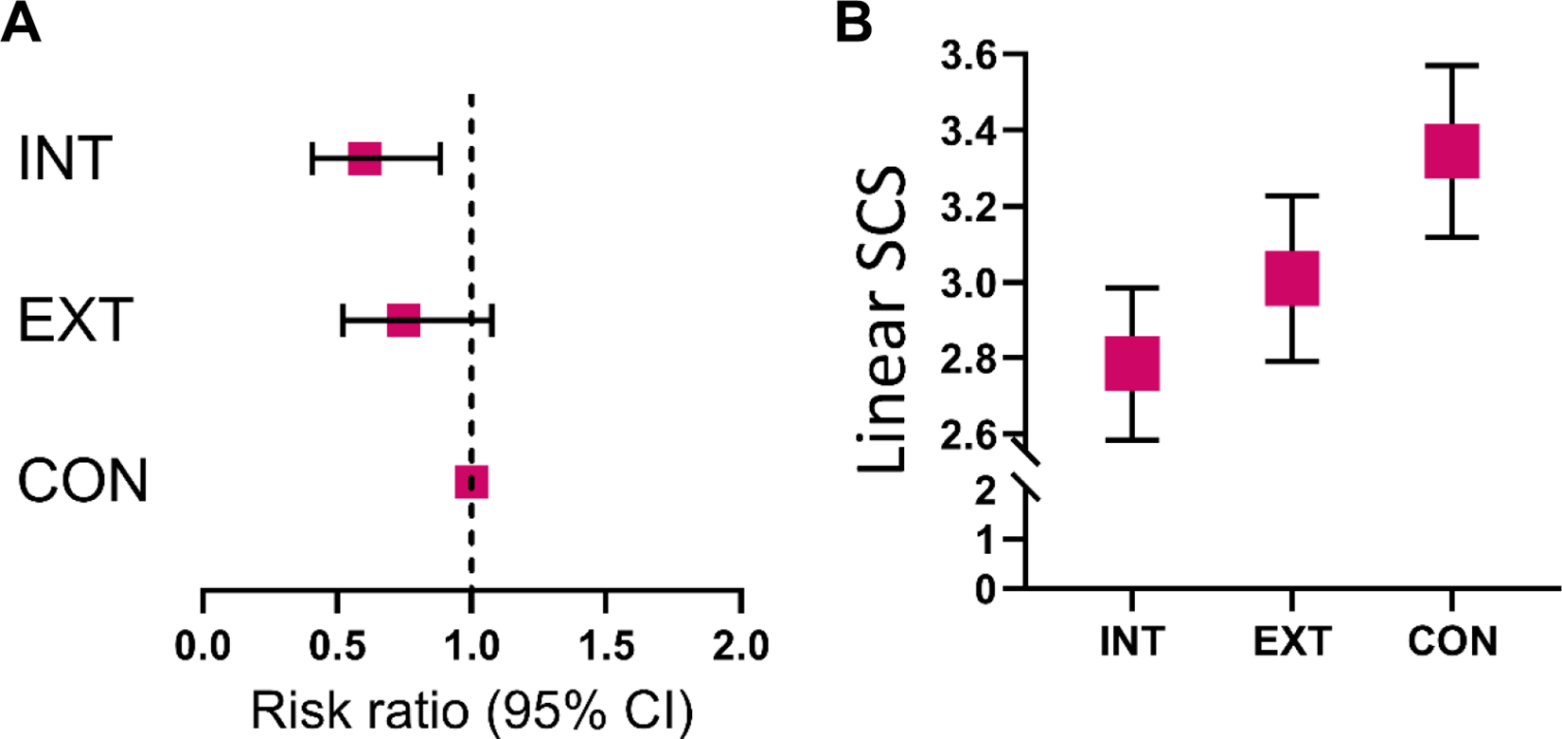
Risk ratios (95% CI) for new IMI risk (A) and mean (95% CI) linear SCS at first DHI test after calving (B) for Holstein cows on a single dairy farm. Cows were randomized at dry-off into 1 of 3 groups in which all functional quarters within a cow were treated in the same manner with (1) 1 tube of intramammary dry-cow antibiotic administered into each teat (CON), (2) 1 tube of intramammary dry-cow antibiotic followed by 1 tube of internal teal sealant administered into each teat (INT), or (3) 1 tube of intramammary dry-cow antibiotic followed by a single dipping of each teat into an external teat sealant (EXT).

Linear SCS at first DHI test after calving differed among groups (*P* = 0.001, Figure 1B). Cows in the CON group (n = 368) had a mean (± SD) LS1 of 3.3 ± 2.2, which was greater than that of INT cows (n = 386; *P* < 0.05) with an LS1 of 2.8 ± 2.0 and not different from that of the EXT cows (n = 374; *P* ≥ 0.05) with an LS1 of 3.0 ± 2.1. First DHI test day milk did not differ among treatment groups (*P* = 0.73) with mean (± SD) yield of 46.3 ± 8.6 kg/d for CON cows, 45.8 ± 8.6 kg/d for INT cows, and 46.2 ± 9.0 kg/d for EXT cows.

No cases of clinical mastitis were diagnosed during the dry period; all documented clinical mastitis cases occurred after calving. The incidence of clinical mastitis ≤30 DIM was 13.0% for the CON group, 13.2% for the INT group, and 11.5% for the EXT group and did not differ among groups (*P* = 0.71). During the dry period, no cows were lost to follow-up. The incidence of culling ≤30 DIM did not differ among groups (*P* = 0.35) and was 5.1% for the CON group, 6.5% for the INT group, and 7.4% for the EXT group.

As expected, cows receiving an INT at dry-off, in addition to antibiotic dry cow treatment, had lower risk of new IMI during the dry period and lower LS at first DHI test after calving. Compared with CON cows, the risk of acquiring a new IMI during dry-off and the first DHI test day was 40% lower. Our results are in accordance with a large body of literature demonstrating the efficacy of INT on the reduction of IMI (Rabiee and Lean, 2013) and suggest that the infusion of INT provides an effective barrier against the invasion of pathogens during the dry period.

Our data provide supporting evidence that cows in group EXT were 25% less likely to acquire a new IMI compared with CON cows; the likelihood that these differences were due chance was 14%. Timms (2001) tested the efficacy of an EXT in a randomized study following a half-udder design in 190 animals. Teats were dipped at dry-off, approximately 10 d prepartum, and then re-dipped as needed until parturition, and a reduction in new IMI at calving of up to 68% was documented. As illustrated by Bradley and Green (2004), the risk of IMI during the dry period is increased shortly after dry-off and before calving. Conversely, the adherence of EXT has been shown to last for an average duration of 6 d ranging from 0 to 15 d (Leslie et al., 1999). We did not document the adherence of the EXT throughout the dry period in the current study. However, we believe that the single application of EXT failed to provide an effective barrier given its reduced performance compared with INT. This is also supported by the manufacturer specifications suggesting 2 applications at dry-off (a second application after the first coating is dry), as well as another series of applications starting at 10 d before calving to achieve maximum protection. We therefore speculate that the reduction in new IMI for group EXT would have been more pronounced if an application before calving had been applied. However, the application of EXT 10 d before calving was omitted for practical reasons.

The absence of differences in cure risk of IMI among groups supports descriptions by other researchers (Huxley et al., 2002; Godden et al., 2003) and is coherent given that none of the sealants contained antimicrobial properties and all cows received an antibiotic dry cow treatment, as previously discussed (Godden et al., 2003). We observed no meaningful differences in clinical mastitis risk within the first 30 DIM after calving. This finding is consistent with those reported by Huxley et al. (2002), but contrasts results from other studies (Godden et al., 2003; Runciman et al., 2010). Differences in study populations, study design, and regions could be factors that account for the observed discrepancies among studies. Specifically, we believe that in the current study population, most new IMI leading to clinical mastitis occurred between calving and 30 DIM, equalizing a possible beneficial treatment effect. This could also explain the lack of differences in first DHI test day milk and culling risk within the first 30 DIM. However, because we have not determined the IMI status at the time of calving, this explanation remains speculative.

Our study had some limitations that the reader should consider. First, the study was conducted on one commercial dairy farm in central New York with a unique management system. For example, the frequency of cows with a LSDRY ≥4 of 33.6% compares to 25.6% of IMI at dry-off in the study by Vasquez et al. (2018). Therefore, our results likely reflect what would happen on dairy operations with similar management and udder health status in this region. However, the generalizability is limited until the results are replicated in other systems and geographic regions. Second, an a priori sample size calculation was not conducted. Although we found meaningful differences in new IMI and LS1 at first DHI test, it is important to mention that the study was not sufficiently powered for the remaining outcome variables. A post hoc power analysis with JMP PRO (v. 17.0, SAS Institute Inc.) using the observed differences among groups CON and INT, the respective cow numbers for each outcome variable, and an α-level of 0.05 yielded power values of 0.89 for new IMI, 0.31 for IMI cure, 0.05 for clinical mastitis ≤30 DIM, 0.14 for culling ≤30 DIM, 0.09 for first DHI test day milk, and 0.58 for LS1. Therefore, future studies with a sufficiently large sample size are needed to investigate the effect of teat sealants on the outcome variables that were underpowered in this study. Last, due to farm management choices, the EXT was applied only once at dry-off for practical reasons. This likely hampered its full potential in providing an effective barrier throughout the entire dry period. Future research is warranted investigating different application regimens of EXT.

In conclusion, our study found that cows dried off with an INT in addition to antibiotic dry cow treatment had a lower LS1 at first DHI test and reduced risk of new IMI than cows dried off with an EXT in addition to antibiotic dry cow treatment or cows dried off using antibiotic dry cow treatment alone. Additionally, we found supportive evidence that cows dried off with an EXT in addition to antibiotic dry cow treatment might have an advantage in reducing new IMI over cows dried off with an antibiotic dry cow treatment alone, with further investigation warranted with a larger sample size. However, there was no difference in risk of clinical mastitis ≤30 DIM, risk of culling ≤30 DIM, or milk yield at first DHI test among groups.
